# Emergence of *Cronobacter sakazakii* in Cases of Neonatal Sepsis in Upper Egypt: First Report in North Africa

**DOI:** 10.3389/fmicb.2020.00215

**Published:** 2020-03-09

**Authors:** Amal A. Elkhawaga, Helal F. Hetta, Naglaa S. Osman, Amal Hosni, Mohamed A. El-Mokhtar

**Affiliations:** ^1^Department of Medical Microbiology and Immunology, Faculty of Medicine, Assiut University, Assiut, Egypt; ^2^Department of Internal Medicine, University of Cincinnati College of Medicine, Cincinnati, OH, United States; ^3^Department of Pediatrics, Children’s Hospital, Faculty of Medicine, Assiut University, Assiut, Egypt; ^4^Department of Clinical Pathology, Faculty of Medicine, Assiut University, Assiut, Egypt

**Keywords:** *C. sakazakii*, powdered infant formula, septicemia, herbs, PCR

## Abstract

**Background and Aim:**

*Cronobacter sakazakii* (*C. sakazakii*) has attracted considerable attention as an emerging neonatal pathogen and has been associated with outbreaks of life-threatening septicemia, necrotizing enterocolitis, and meningitis in neonates and infants globally. No data about the role of *C. sakazakii* as a cause of neonatal sepsis in North Africa is availale. Herein, we aimed to study the incidence of *C. sakazakii* in cases of neonatal sepsis, its distribution in different food samples in Egypt, antimicrobial profile, and the ability of the strains to form biofilms.

**Methods:**

A total of 100 positive blood cultures from cases of neonatal sepsis admitted to the neonatal ICU at Assiut University Children’s Hospital, Egypt, were analyzed. In addition, 1,100 food samples, including 400 powdered infant formula (PIF), 500 herbs, and 200 water samples were screened for the presence of *C. sakazakii*. We evaluated the antimicrobial profile and the ability of the strains to form biofilms.

**Results:**

*Cronobacter sakazakii* was detected in 12 out of 100 cases of neonatal sepsis. The organism was also isolated from PIF, herbs, and water in percentages of 17.5, 9.2, and 7.5%, respectively. Regarding the antimicrobial sensitivity, all strains were resistant to ampicillin, amoxicillin, ampicillin/sulbactam, clindamycin, cephalothin, and cephalexin. In addition, *C. sakazakii* strains showed the ability to form biofilms, but with variable degrees of cell density.

**Conclusion:**

We reported, for the first time, cases of neonatal sepsis caused by the emerging life-threatening pathogen *C. sakazakii* in Egypt. The organism was also detected in contaminated PIF, herbs, and water, which may be sources of infection for neonates, especially in countries where natural herbs are widely used as an alternative medicine. Finally, collective efforts by the parents, manufacturers of PIF, and healthcare personnel are essential to prevent serious infections caused by *C. sakazakii*, particularly in infants.

## Introduction

The *Cronobacter* genus is a member of the family Enterobacteriaceae that includes seven species, of which *Cronobacter sakazakii* is the main species linked to life-threatening infections in infants and immunocompromised adults (Feeney et al., 2014). It is a Gram-negative, motile, non-spore-forming, facultative anaerobe. *C. sakazakii* is generally resistant to osmotic stress and dryness; therefore, it could be detected in stored powdered infant formula (PIF) even after 2.5 years of storage (Bai et al., 2019). Moreover, *C. sakazakii* was detected in a wide variety of environmental samples, food, and herbs (Mayor, 2004; Baumgartner et al., 2009).

*Cronobacter sakazakii* is seriously implicated in cases of neonatal septicemia, necrotizing enterocolitis, and meningitis (Chenu and Cox, 2009; Holı and Forsythe, 2014). Neonates with sepsis usually present with non-specific features. Diagnosing the etiology and detecting the causative pathogen are crucial to improve outcomes (The Young Infants Clinical Signs Study Group, 2008; Voller and Myers, 2016). *Cronobacter* spp. have also been associated with cases of conjunctivitis, aspiration pneumonia, diarrhea, wounds, abscesses, and urinary tract infections (Tsai et al., 2013). Flores et al. (2011) reported nosocomial infections caused by *Cronobacter* spp. *C. sakazakii* was specified by the International Commission on Microbiological Specifications for Food (ICMSF, 2002) as “severe risk for a restricted population, representing a threat of death or chronic consequence of long duration” (Feeney et al., 2014).

The precise pathogenesis of *C. sakazakii* remains not fully explained. However, the outer membrane protein A (OmpA) is considered a potential virulence factor. It is required for the binding and invasion of brain endothelial cells as necessary steps for the development of meningitis. It was recorded that the OmpA region is appropriate for the identification of *Cronobacter* spp. with higher specificity than internal transcribed spacer (ITS) sequences, 16S rRNA, and *gluA* and *gluB* genes (Singamsetty et al., 2008; Fei et al., 2015). Other plasmid-associated genes such as the *Cronobacter* plasminogen activator, filamentous hemagglutinin, and genes responsible for iron acquisition were reported in *C. sakazakii* (Franco et al., 2011). Besides the ability to form biofilms and to resist environmental stresses, antibiotics and high-level disinfectants contribute to the pathogenic potential of *C. sakazakii*.

This study aimed to isolate *C. sakazakii* from cases of neonatal sepsis admitted to the neonatal ICU of Assiut University Children’s Hospital, and from PIF, herbs, and water samples randomly collected from Assiut City in Egypt, to evaluate the antimicrobial profile and the ability of the strains to form a biofilm.

## Materials and Methods

### Ethical Consideration

The study was approved by the Medical Ethics Committee, Faculty of Medicine, Assiut University (IRB no. 17300296). Informed written consent was obtained from all parents or guardians of the neonates before enrollment in the study.

### Study Design and Population

This study was a cross-sectional, hospital-based, descriptive study. Samples were collected from preterm infants with neonatal sepsis. Neonates were admitted to Assiut University Children’s Hospital, Egypt, in a period of 1 year from December 15, 2017 to December 14, 2018.

The study included 100 preterm neonates of <37 weeks gestational age and aged from 0 to 28 days because the admission criteria of the neonatology unit of Assiut University Children’s Hospital include only neonates who are less than 4 weeks of age; infants older than 28 days are transferred to the pediatric ICU.

Neonatal sepsis was clinically suspected based on the presence of any sign of the following; convulsions, lethargy, change in the feeding pattern, tachypnea (respiratory rate, >60/min), signs of respiratory distress (marked chest indrawing), grunting, cyanosis, fever or hypothermia, and elevated C-reactive protein (CRP) (The Young Infants Clinical Signs Study Group, 2008). For these neonates, blood cultures were collected and analyzed. Neonates were excluded from the analysis if the diagnosis of sepsis was presumptive and blood cultures were negative.

### Data and Sample Collection

A total number of 100 blood cultures were collected from cases of neonatal sepsis admitted to the neonatal ICU at Assiut University Children’s Hospital. The number of samples was limited by the consent rate. Neonates were subjected to complete clinical examination (temperature, respiration, color, presence of lethargy, or any neurological troubles and change in feeding pattern). Laboratory investigations, including complete blood count (CBC) and CRP evaluation, were carried out as shown in Table 1. Blood cultures were collected to be tested for the presence of bacterial infections, including *C. sakazakii*. Samples had been collected for general screening purposes. Approximately 1–4 ml of blood was aseptically collected from the peripheral vein and inoculated directly into BacT/ALERT blood culture bottles (bioMérieux, Marcy l’Etoile, France), which was monitored using the BacT/ALERT 3D instrument (bioMérieux, Marcy l’Etoile, France). The bottles were incubated at 37°C for 7 days, and positive specimens were inoculated onto Brilliance *C. sakazakii* chromogenic agar (DFI, Oxoid, United Kingdom), MacConkey agar, and blood agar and incubated for 24 h at 36°C. Also, a sample was streaked onto trypticase soya agar, which was incubated at 25°C. Suspected colonies were further confirmed using VITEK 2 automated microbiology system (VITEK 2 GN ID card, bioMeriéux’s) and subtyped using real-time PCR (RT-PCR), as described in the next sections.

**TABLE 1:** Demographic and clinical data of the study population.

Variable	Number of cases (%)
Male gender	67 (67%)
Birth weight (g)	
Median (range)	2,000 (1,000–3,000)
Residence	
Rural	78 (78%)
Urban	22 (22%)
Cesarian section	83 (83%)
Low Apgar score at 1 min	33 (33%)
Low Apgar score at 5 min	12 (12%)
Central line insertion	97 (97%)
Maternal risk factors	
Preeclampsia	8 (8%)
Premature rupture of membranes	5 (5%)
Fever	7 (7%)
Antepartum hemorrhage	2 (2%)
Clinical manifestations	
Poor oral intake	77 (77%)
Fever >37.8°C	87 (87%)
Hypothermia <36°C	13 (13%)
Jaundice	31 (31%)
Eye discharge	9 (9%)
Skin rash	11 (11%)
Respiratory distress	88 (88%)
Apnea	19 (19%)
Pneumonia	33 (33%)
Diarrhea	28 (28%)
Elevated CRP	100 (100%)
Total leukocytic count (cells/μl)	
Mean ± SD	22.97 ± 11.38
Median (range)	23.85 (2.9–49.0)
Absolute neutrophilic count (<1,000 cells/μl)	8 (8%)
Platelet count (<100 cells/μl)	23 (23%)

*The Apgar score comprises five components: heart rate, respiratory effort, muscle tone, reflex irritability, and color, each of which is given a score of 0, 1, or 2. A low Apgar score means a score less than 7 at 1 and 5 min (American Academy of Pediatrics, 2006; Cheng et al., 2013). Jaundice is a yellow discoloration of the sclera. Respiratory distress symptoms include tachypnea, grunting, nasal flaring, and intercostal retractions. Pneumonia is evident on chest X-ray.*

In addition, different food samples were analyzed for the presence of *C sakazakii*, including (Iversen and Forsythe, 2007) 500 herbs and 200 water samples. PIF and herbs were randomly collected from local supermarkets, pharmacies, herbal shops, and Assiut University Children’s Hospital in Assiut City. Herb samples (*n* = 500) included the following types: 50 anise, 50 licorice, 50 green tea, 50 mint, 50 fenugreek, 50 caraway, 50 hibiscus, 50 herbal tea, 50 chamomile, and 50 thyme. Regarding water samples (*n* = 200), they were randomly obtained from the following sources: 50 bottled water, 50 underground water, 50 river water, and 50 tap water. These samples were collected in the same time frame of the study.

### Isolation and Identification of *Cronobacter* spp.

Isolation was carried out basically according to the U.S. Food and Drug Administration isolation and enumeration method (U.S. Food and Drug Administration, 2002). Briefly, 100 g of PIF was added to 900 ml buffered peptone water (BPW) to refresh stressed cells, then gently mixed and incubated at 36°C for 24 h. Ten milliliters of the pre-enrichment mixture was added into 90 ml of Enterobacteriaceae enrichment (EE) broth and further incubated at 36°C for 24 h. Then, the mixture was centrifuged at 3,000 × *g* for 10 min and the pellet was suspended into 1 ml of sterile phosphate-buffered saline.

**FIGURE 1:**
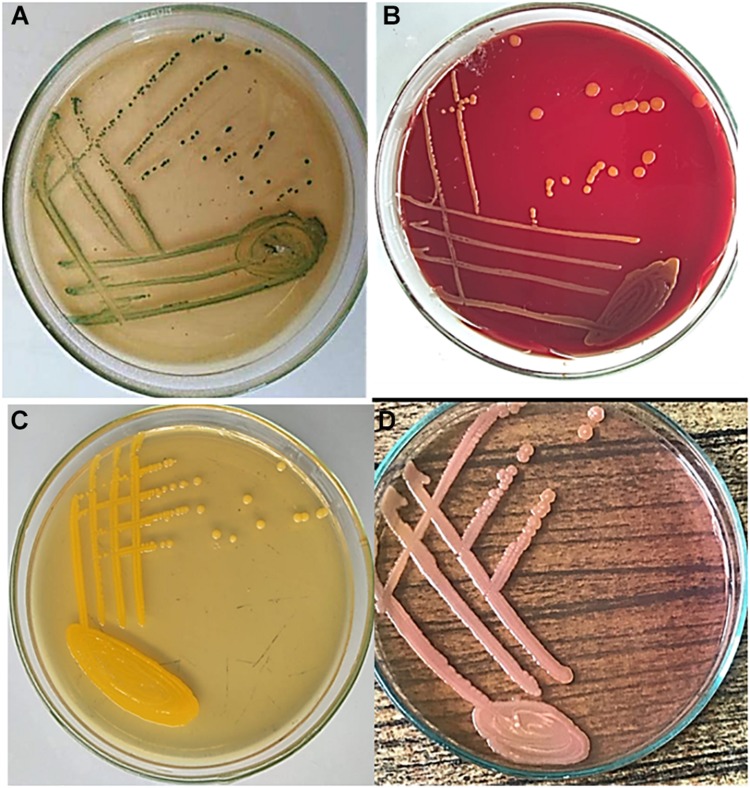
Morphological appearance of *Cronobacter sakazakii* colonies. Samples were inoculated on **(A)** Brilliance *C. sakazakii* chromogenic agar showing greenish colonies; **(B)** blood agar showing non-hemolytic colonies; **(C)** trypticase soya agar (TSA) showing flat yellow colonies; and **(D)** MacConkey agar showing pink lactose-fermenting colonies. Plates were incubated for 24 h at 36°C, except for trypticase soya agar which was incubated at 25°C. TSA was not supplied with 5% sheep blood.

Regarding herbs, 10 g of each herb sample was mixed with 90 ml sterile distilled water and was incubated at 36°C overnight. Then, 1 ml of pre-enrichment mixtures was inoculated into 9 ml EE broth and incubated at 36°C overnight. For water samples, 100 ml was filtered through a 0.45-μm cellulose nitrate membrane filter by a filtration apparatus (Altmann Analytic, Munich, Germany). The filter paper was inoculated into the EE broth and incubated at 36°C for 24 h (Chen et al., 2016). For phenotypic identification, 100 μl of the selective EE broth was streaked onto Brilliance *C. sakazakii* chromogenic agar (DFI, Oxoid, United Kingdom), MacConkey agar, and blood agar and incubated for 24 h at 36°C. Also, a sample was streaked onto trypticase soya agar which was incubated at 25°C. Suspected green colonies on Brilliance *C. sakazakii* chromogenic agar were further confirmed using the VITEK 2 automated microbiology system (VITEK 2 GN ID card, bioMerieux’s). Figure 1 shows the morphological appearance of *C. sakazakii* colonies on the different culture media.

### Identification of *C. sakazakii*

Subtyping of the *Cronobacter* strains was carried out by RT-PCR targeting the *OmpA* gene of *Cronobacter* spp. using the following primers: ESOMP5-F: 5′-GGTGAAGGATTTAACCGTGAACTT-3′ and ESOMP5-R: 5′-GCGCCTCGTTATCATCCAAA-3′ (Invitrogen, United States). This was followed by high resolution melting (HRM) analysis in the 7500 Fast Real-Time PCR System (Applied Biosystems, United States), as previously described (Cai et al., 2013). Briefly, genomic DNA of the isolated colonies was extracted using the Genejet DNA extraction kit (Thermo Fisher Scientific, United States). PCR reaction was carried out in a total volume of 20 μl, which consisted of 1× Fast-plus Evagreen qPCR master Mix (Bio-Rad, United States), dNTPs, MgCl_2_, 0.3 μM of each primer, and 4 μl genomic DNA as a template. The PCR program consisted of an initiation denaturation at 95°C for 4 min, then 40 cycles of denaturation at 95°C for 15 s, and annealing at 60°C for 15 s, followed by extension at 72°C for 20 s. After the PCR amplification, a melting curve analysis was performed to ensure the specificity of the RT-PCR amplification curves and to differentiate between the *Cronobacter* spp. Nuclease-free water was included as the no template control (NTC). *C. sakazakii* (ATCC 29544) and *Cronobacter muytjensii* (ATCC 51329) were used as the positive controls. To assure the specificity of the assay, *Franconibacter helveticus* (LMG 23732), *Franconibacter pulveris* (LMG 24057), and *Siccibacter turicensis* (LMG 23730) were included in the reactions (purchased from Belgian Coordinated Collections of Microorganisms). The HRM profiles of all isolates produced distinct *T*_*m*_ peaks which were clustered into the same group with the reference *C. sakazakii* (ATCC 29544) and was represented by a melting peak at *T*_*m*_ = 79.2 ± 0.05°C. In contrast, no positive fluorescence signals were obtained from the non-*Cronobacter* strains or the non-template control sample (Cai et al., 2013).

### Antibiotic Sensitivity Test

Isolated colonies of *C. sakazakii* were examined using the modified Kirby–Bauer disc diffusion method. Results of the antibiotic sensitivity were interpreted according to the regulations of the Clinical and Laboratory Standards Institute (CLSI, 2018). The tested antibiotics included ampicillin (10 μg), amoxicillin (25 μg), ampicillin-sulbactam (20 μg), tetracycline (30 μg), gentamycin (10 μg), erythromycin (15 μg), clindamycin (2 μg), tobramycin (10 μg), chloramphenicol (30 μg), cefoxitin (30 μg), cefuroxime (30 μg), cephalexin (10 μg), cephalothin (30 μg), cefadroxil (30 μg), cefoperazone (75 μg), ciprofloxacin (5 μg), ceftriaxone (30 μg), norfloxacin (10 μg), levofloxacin (10 μg), and imipenem (10 μg).

### Evaluation of Biofilm Formation by *C. sakazakii*

The ability of *C. sakazakii* to adhere and to form biofilms in a 24-well plate was done using the microtiter plate assay. Overnight cultures of *C. sakazakii* in a brain heart infusion broth were seeded into 24-well microtiter plates and incubated at 37°C for 18 h. The cultures were removed and, using sterile distilled water, the wells were rinsed three times to remove unattached cells and dried overnight. Crystal violet aqueous solution (1%) was added into the wells and left for 15 min, followed by rinsing the wells using sterile distilled water and dried overnight. Of 33% glacial acetic acid, 300 μl was added into the wells to dissolve the remaining crystal violet and the absorbance was measured at a wavelength of 570 nm, which corresponds to the mass of the biofilm in the well (Yusof et al., 2017). The average optical density (OD) values were calculated for all the tested strains and negative controls. The cutoff value (ODc), defined as three standard deviations (SDs) above the mean OD of the negative control, was calculated as follows: ODc = average OD of negative control + (3 × SD of negative control). The final OD value of a tested strain is expressed as the average OD value of the strain reduced by the ODc value (OD = average OD of a strain−ODc). Strains were divided into the following categories: no biofilm producer if OD ≤ ODc, weak biofilm producer if ODc < OD ≤ 2 × ODc, moderate biofilm producer if 2 × ODc < OD ≤ 4 × ODc, and strong biofilm producer if 4 × ODc < OD (Stepanoviæ et al., 2007).

### Statistical Analysis

Statistical analyses were performed using the Statistical Package for Social Sciences, version 16.0 (SPSS Inc., Chicago, IL, United States). Data are represented as the mean ± SD for continuous variables and as percentages for categorical variables.

## Results

The demographic and clinical characteristics of the neonates are summarized in Table 1. *C. sakazakii* was isolated from 12 out of 100 culture-proven sepsis, giving an incidence rate of 12%. After receiving multiple symptomatic treatments, the clinical signs improved in eight neonates out of the 12 *C. sakazakii* cases. However, the mental and physical status of the other two cases were markedly impaired and the remaining two cases died after 2 weeks of admission.

The remaining 88 positive blood cultures revealed other bacterial agents. *Klebsiella pneumoniae* was the most common microorganism causing neonatal sepsis (22%), followed by *Acinetobacter baumannii* (16%), coagulase-negative staphylococci (15%), methicillin-resistant *Staphylococcus aureus* (12%), *Pseudomonas aeruginosa* (10%), and *Streptococcus pneumoniae* (6%). *Candida albicans* was detected in 7% of the cases.

In addition to the clinical cases, 1,100 food samples, including infant formulas, herbs, and water samples, were tested for the presence of *Cronobacter* spp. Table 2 summarizes the categories of herbs and water samples analyzed for the presence of *Cronobacter* spp. *C. sakazakii* was detected in 70 of 400 (17.5%) PIF samples. We also tested whether herbs were contaminated with *C. sakazakii* since herbs are of common use for neonates. Among 500 herb samples, 45 (9.2%) were positive for *Cronobacter* spp. The highest percentages of *Cronobacter* spp. were found in licorice (17/50, 26%) and anise (17/50, 26%). We did not detect *C. sakazakii* in hibiscus, fenugreek, and thyme. Moreover, 15 isolates were recovered from 200 water samples. The highest percentage of *Cronobacter* spp. was detected in underground water (8/50, 16%).

**TABLE 2:** Prevalence of *Cronobacter sakazakii* in the analyzed samples.

Types of samples	Number of *C. sakazakii* isolates (%)
Blood cultures	12/100(12%)
Powdered infant formula (PIF)	70/400(17.5%)
From neonatal ICU	46/200(23%)
Used PIF containers	21/100(21%)
Unopened PIF containers	3/100(3%)
Herbs	46/500(9.2%)
Anise	17/50(26%)
Licorice	13/50(34%)
Green tea	4/50(8%)
Mint	4/50(8%)
Fenugreek	0/50(0%)
Caraway	2/50(4%)
Herbal tea	5/50(10%)
Hibiscus	0/50(0%)
Chamomile	1/50(2%)
Thyme	0/50(0%)
Water	15/200(7.5%)
Bottled water	0/50(0%)
Tap water	4/50(8%)
Underground water	8/50(16%)
River water	3/50(6%)

All isolated strains were resistant to ampicillin, amoxicillin, ampicillin/sulbactam, clindamycin, cephalothin, and cephalexin. On the other hand, they showed 100% sensitivity for levofloxacin, tetracycline, imipenem, and chloramphenicol. The sensitivity values to other antibiotics were 94% for erythromycin, 95.2% for gentamycin, 96.4% for tobramycin, and 14.2% for cefadroxil (Table 3).

**TABLE 3:** Antibiotic susceptibility profile of the isolated *Cronobacter sakazakii* strains.

Antibiotics	Sensitivity of *C. sakazakii* (*N* = 84)	
	Sensitive *N* (%)	Resistant *N* (%)
Ampicillin (10 μg)	0 (0)	84 (100)
Amoxicillin (25 μg)	0 (0)	84 (100)
Ampicillin/sulbactam (20 μg)	0 (0)	84 (100)
Tetracycline (30 μg)	84 (100)	0 (0)
Gentamycin (10 μg)	80 (95.2)	4 (4.7)
Erythromycin (15 μg)	79 (94)	15 (17.8)
Clindamycin (2 μg)	0 (0)	84 (100)
Tobramycin (10 μg)	81 (96.4)	3 (3.5)
Chloramphenicol (30 μg)	84 (100)	0 (0)
Cefoxitin (30 μg)	84 (100)	0 (0)
Cefuroxime (30 μg)	0 (0)	84 (100)
Cephalexin (10 μg)	0 (0)	84 (100)
Cephalothin (30 μg)	0 (0)	84 (100)
Cefadroxil (30 μg)	12 (14.2)	72 (84.5)
Cefoperazone (75 μg)	84 (100)	0 (0)
Ceftriaxone (30 μg)	84 (100)	0 (0)
Ciprofloxacin (5 μg)	84 (100)	0 (0)
Norfloxacin (10 μg)	84 (100)	0 (0)
Levofloxacin (10 μg)	84 (100)	0 (0)
Imipenem (10 μg)	84 (100)	0 (0)

**N* is the total number of isolated *C. sakazakii* strains.*

All isolates were evaluated for their ability to form a biofilm using the microtiter plate method. As shown in Table 4, all strains were able to form biofilms. Around half of the isolated strains were able to form strong biofilms and the other half able to form moderate ones.

**TABLE 4:** Biofilm formation by isolated *Cronobacter sakazakii*.

Source	Strong biofilm producer (OD_570_ > 2)	Moderate biofilm producer (1 < OD_570_ > 2)	Weak biofilm producer (0.5 < OD_570_ > 1)	NO biofilm formation (OD_570_ < 0.5)
Cases of neonatal sepsis (*n* = 12)	7	5	–	–
PIF samples (*n* = 70)	37	33	–	–
Herbs samples (*n* = 46)	23	23	–	–
Water samples (*n* = 15)	10	5	–	–

## Discussion

*Cronobacter sakazakii* is considered an opportunistic pathogen of great concern to neonatal health not only in developing countries but also worldwide (Feeney et al., 2014). Neonatal sepsis is the third leading cause of neonatal mortality, defined as blood infections that occur in infants ≤28 days (4 weeks), and exemplifies a significant health burden especially in very low-birth-weight infants (<1,500 g) and preterm infants (<34 weeks of gestation) (Zea-Vera and Ochoa, 2015). Concerning our protocol of isolation, our methods followed the classical U.S. Food and Drug Administration (2002) for the isolation of *C. sakazakii* from dehydrated PIF. We wanted to change our protocol to follow the revised FDA method, but some samples were already analyzed using the old protocol. We also wanted to reanalyze the samples according to the revised protocols, but unfortunately, we did not have enough samples to complete the reanalysis. Therefore, to avoid any bias in our analysis, we decided to process all samples using the same conditions. One of the limitations of the EE broth is its lower ability to recover *Cronobacter* isolates which are heat-, acid-, alkaline-, or desiccation-stressed. Moreover, the EE broth was inferior in detecting some *Cronobacter* strains and may support the growth of other competing Enterobacteriaceae (Gurtler and Beuchat, 2005; Iversen and Forsythe, 2007; Al-Holy et al., 2008, 2011). Therefore, new methods have been developed based on chromogenic media to improve the isolation and detection of *Cronobacter* from different samples (Lampel and Chen, 2009). However, the differential selective media showed a lower ability in supporting the resuscitation and colony formation by stressed cells (Gurtler and Beuchat, 2005). Also, some *Cronobacter* strains did not produce the typical colored colonies on these media (Iversen and Forsythe, 2007). Therefore, our identification protocol was not based only on the appearance of green colonies on Brilliance *C. sakazakii* chromogenic agar; however, the suspected colonies were further confirmed using the VITEK 2 system. Of note is that all our isolates produced the typical green colonies on the Brilliance *C. sakazakii* chromogenic agar.

Herein is the first report of *C. sakazakii* infection in cases of neonatal sepsis in North Africa. Out of the 100 blood cultures obtained in the present study, 12 cases (12%) were positive for *C. sakazakii*, which represents a higher rate when compared with the reported rate in the United States, which was approximately 1 in 100,000 infants and increased to approximately 1 in 11,000 infants of less than 1,500 g birth weight (Stoll et al., 2004). Data from six FoodNet sites in the United States revealed that the highest percentage of invasive *Cronobacter* infections occurred among infants (6/22, 27%) and children 1–4 years of age (5/23, 22%) (Patrick et al., 2014). In the Czechia, Holy et al. studied the incidence of *Cronobacter* spp. collected for a period of 7 years (2005–2011) from different pathological samples. They reported that a high recovery of *Cronobacter* spp. (63.7% of *Cronobacter*) was from children 1–14 years of age (Holı and Forsythe, 2014). The study was further extended by Alsonosi et al. (2015), who genotyped 51 *Cronobacter* strains from clinical isolates that have been collected in a survey of *Cronobacter* during a 6-year period (2007–2013) and reported *C. sakazakii* (65%) to be the major detected species, followed by *Cronobacter malonaticus* (33%). Lepuschitz et al. (2019) reported a low *C. sakazakii* frequency, where only 11 of the 24 (45.8%) participating countries in Europe submitted *C. sakazakii* isolates, which was attributed to the imperfect detection system.

Generally, preterm infants are more susceptible to infections than any other age (Hunter and Bean, 2013). This may be explained by the fact that the transplacental passage of antibodies peaks during the third trimester. Therefore, most preterm infants have significantly reduced humoral immune responses (Cui et al., 2017).

The prevalence rates of *C. sakazakii* in the PIF in our study are generally higher than most rates at which *Cronobacter* spp. were detected by investigations conducted in other countries. The prevalence of *Cronobacter* spp. differs according to the type of food and according to the geographical distribution. Alarmingly, a recent study reported that the incidence rates for the detection of *C. sakazakii* were 10 and 35% of the examined PIFs produced in Singapore and Chile, respectively (Parra-Flores et al., 2018). In China, Pan et al. (2014) examined *Cronobacter* spp. contamination in commercial PIFs and follow-up formulas. In this study, *Cronobacter* spp. were detected in 49 of the 399 samples. The isolation rates from PIFs and follow-up formulas were 11.5 (19/165) and 12.8% (30/234), respectively. The isolates included 48 *C. sakazakii* and only one *C. malonaticus*. Similar high rates were also observed in Jordan (12% of infant foods and drinks), where *C. sakazakii* was the only species isolated from the analyzed products (Chap et al., 2009). In Netherlands, *C. sakazakii* was detected in 14.2% of samples (Muytjens et al., 1988). Other published studies have detected *Cronobacter* spp. in 9.3% in the United Kingdom (Chap et al., 2009), 6.0% in South Korea (Kim et al., 2011), and 6.7% in Canada (Nazarowec-White and Farber, 1997a). A meta-analysis of studies reported between 2008 and 2014 for the prevalence of *Cronobacter* spp. in animal- and plant-related food samples showed that *Cronobacter* spp. could be isolated from 19% of the plant-related food samples, while 5.7% of the animal-related food was contaminated with the bacteria (Sani and Odeyemi, 2015). This high rate of positivity should lead to better control by infant formula manufacturers and healthcare authorities to avoid contamination of this dangerous bacteria.

Although it was shown that *C. sakazakii* would not survive the pasteurization process, it is possible that contamination may occur during the addition of the dry components, such as minerals and vitamins, to the PIF before packaging or due to the poor hygienic practices during the production process (Nazarowec-White and Farber, 1997b; Drudy et al., 2006). *C. sakazakii* is the dominant species of *Cronobacter* spp. isolated from PIF and environmental samples and can infect infants and adults, respectively (Fei et al., 2015).

In Egypt, some herbs like anise, caraway, mint, fenugreek, and chamomile are widely used and are supposed to relieve gastrointestinal disturbances in infants (Al-Nabulsi et al., 2009; John and Shantakumari, 2015). *C. sakazakii* was detected in 9.2% of the tested herb samples, which is consistent with the results obtained by Aksu et al. (2016, 2018), who reported in their study that 14% of spice and herb samples tested in Turkey were positive for *Cronobacter* spp. and that the predominance was for *C. sakazakii*. Similarly, Garbowska et al. (2015) isolated *Cronobacter* spp. from 16.7% of tested herbs in Poland. Turcovský et al. (2011) reported a higher prevalence of *Cronobacter* spp. in plant-originated foods (31.29%) than in animal origin foods (6.15%). Iversen and Forsythe (2004) concluded that the natural habitat of *Cronobacter* spp. could be plant material because they could isolate these bacterial strains from plant-related products such as dry herbs and spices.

In our study, *C. sakazakii* was detected at a high rate in licorice (34%) and anise (26%), but we could not detect *C. sakazakii* in fenugreek, hibiscus, or thyme. Fenugreek (*Trigonella foenum-graecum* L.) is one of the most promising ancient medicinal herbs especially in the Mediterranean region and Asia. It contains different alkaloids, flavonoids, and saponins, which have antibacterial activity and also enhance antioxidant capacity (Dixit et al., 2005; Sharma et al., 2017). In addition, several studies revealed that *Hibiscus rosa-sinensis* contains compounds with antimicrobial properties such as cyanidin, quercetin, hentriacontane, calcium oxalate, thiamine, riboflavin, niacin, and ascorbic acid (Patel et al., 2012). Regarding thyme (*Tymus vulgaris*), Boskovic et al. (2015) reported that it contains carvacrol and thymol, which have strong antibacterial activity achieved by the disintegration of the outer membrane of the Gram-negative bacteria.

*Cronobacter sakazakii* was isolated from 15 out of 200 (7.5%) water samples. The prevalence was highest in underground water, followed by tap water and river water. Fei et al. (2018) isolated five strains of *C. sakazakii* from 100 drinking water samples. Cui et al. (2017) and Fei et al. (2018) reported that drinking water was the primary source of *Cronobacter* spp. isolates. Therefore, it is recommended to prevent contaminating PIF with *Cronobacter* spp. in order to avoid harmful infections. According to the WHO instructions, hands should be washed thoroughly and feeding equipment should be adequately sterilized. Water should be boiled and not left for more than 30 min after boiling. Feed that is not consumed within 2 h should be discarded (World Health Organization [WHO], 2007).

According to the results obtained in the present study, all *C. sakazakii* strains showed biofilm formation, with variable degrees. Generally, *Cronobacter* spp. have variable abilities to form biofilms (Wang et al., 2018). Previous studies have shown that *C sakazakii* can attach to enteral feeding tubes within only 2 h of exposure. Moreover, *C sakazakii* was able to bind to different surfaces like latex, polycarbonate, and silicon (Iversen et al., 2004). *C. sakazakii* in biofilms are protected by secreted extracellular polymeric substances that form a protective shield from desiccation tolerance and abiotic stresses (Flemming et al., 2016; Lebre et al., 2017). Another problem associated with the ability of *Cronobacter* to form biofilms is that it renders them more resistant to antibiotics as well as high-level disinfection (Kalyantanda et al., 2015). The ability of *Cronobacter* spp. to attach to infant feeding equipment may render these surfaces reservoirs and sources of infection for the infants.

## Conclusion

*Cronobacter sakazakii* is an important cause of neonatal sepsis in Egypt. The organism was also detected in a range of other foods, including PIF, herbs, and water, which has raised the most concern since these foods may represent a potential source of infection to the infants, in particular in countries where natural herbs are widely used as alternative medicine. The biofilm-forming abilities and the resistance to different antibiotics necessitate future active surveillance to determine the incidence of laboratory-confirmed infections and contamination of food or food products with *Cronobacter* spp. This will improve our understanding of the public health effects caused by this pathogen and will eventually minimize its infections in susceptible individuals. Finally, collective efforts by parents, manufacturers of PIF, and healthcare personnel are essential to prevent serious infections caused by *C. sakazakii*, particularly in infants.

## Data Availability Statement

All datasets generated for this study are included in the article/supplementary material.

## Ethics Statement

The studies involving human participants were reviewed and approved by the Assiut University, Faculty of Medicine, Medical Ethics Committee (IRB no: 17300296). Informed written consent was taken from all parents or guardians of the neonates before recruitment in the study. Written informed consent to participate in this study was provided by the participants’ legal guardian/next of kin.

## Author Contributions

All authors contributed equally in the conception of the research idea, methodology design, performed data analysis and interpretation, prepared the manuscript for publication, read and approved the final manuscript.

## Conflict of Interest

The authors declare that the research was conducted in the absence of any commercial or financial relationships that could be construed as a potential conflict of interest.

## Abbreviations

*C. sakazakii*: *Cronobacter sakazakii*
PIF: powdered infant formula.
